# The effect of COVID-19 pandemic on the fruit juice industry: Insights from Türkiye

**DOI:** 10.1016/j.heliyon.2024.e29406

**Published:** 2024-04-09

**Authors:** Berkay Keskin

**Affiliations:** Ankara University, Faculty of Agriculture, Department of Agricultural Economics, Ankara, Türkiye

**Keywords:** COVID-19, Fruit juice, Industry, Export, Türkiye

## Abstract

The COVID-19 pandemic has caused major problems in many areas of the world and has deeply affected many sectors and industries. The food and beverage industry is one of the industries that has been severely affected by the COVID-19 pandemic. This study aims to explore the impact of the pandemic on the Turkish fruit juice industry and the attitudes and behaviors of companies. In addition, the study seeks to identify the changes in the industry caused by the pandemic and discuss its long-term effects. The material used for the study consists of the data obtained from the surveys conducted among the companies producing fruit juices in Türkiye. The results show that the pandemic had a significant impact on companies' logistics activities, while supply activities were moderately affected. However, the effects of the pandemic on exports, sales, production, total profit, and R&D activities were comparatively limited. When comparing the pre-pandemic period with the first year of the COVID-19 pandemic, it was observed that some companies in the industry experienced declines in production, sales, exports, and overall profit, whereas others experienced increases in these metrics. These variations were not associated with company size or length of operation, suggesting that the impact of the pandemic on individual companies was unique. Interestingly, some companies reported positive effects from the pandemic, such as increased demand for certain products, new export markets, improved food safety practices, new suppliers and improved crisis management skills. However, the fruit juice industry in Türkiye was found to be vulnerable in the areas of logistics and supply chain management. The study suggests that companies should strengthen supply chain management, improve stock management, and create online marketing plans to reduce potential problems in future crises. Additionally, it contributes to the development of strategies to mitigate the impact of future pandemics or bottlenecks that may emerge in the future, thereby promoting the efficiency and sustainability of the fruit juice industry as a whole.

## 1 Introduction

The COVID-19 pandemic is the greatest global crisis of the 21st century, affecting people in unprecedented ways. Millions of deaths have been reported worldwide and the pandemic has led to the implementation of curfews, travel bans, and work-from-home mandates for many. The COVID-19 pandemic has caused unprecedented disruption worldwide, resulting in halted industries, disrupted logistics, and panic-buying becoming a common phenomenon. The food and beverage industry has been one of the sectors most severely affected. Therefore, researchers have focused on developing strategies to mitigate the impact of the pandemic on this industry. Addressing the consequences of the pandemic on food and beverage has become a crucial area of concern.

While many studies in the literature examine the impact of crisis periods on agricultural and food companies, as well as how company managers react to such crises [[1], [2], [3], [4], [5]], the COVID-19 pandemic has garnered more attention from researchers due to its unprecedented nature in recent times. Several studies have investigated the effects of the pandemic on the food and beverage industries. Some of these studies have focused on the industry as a whole [[6], [7], [8], [9], [10]], while others have examined specific sectors, including the brewery industry [[11], [12], [13]], dairy industry [[14], [15], [16], [17], [18], [19], [20]], fish industry [[21], [22], [23]], fruit and vegetable industries [24,25], grain industry [26], meat industry [[27], [28], [29], [30], [31], [32]], oil industry [33,34], poultry industry [[35], [36], [37], [38]], shrimp industry [39], sugar industry [40], and wine industry [[41], [42], [43], [44]], revealing the pandemic's effects on these industries. However, the literature on the impact of COVID-19 on the fruit juice industry is very limited, and despite its significance, there is a considerable research gap in this area. This study aims to fill this research gap by providing a comprehensive understanding of the effect of COVID-19 on the fruit juice industry.

The fruit juice industry is significant for several reasons. Fruit juices are popular beverages that contain vitamins, minerals, trace elements, flavonoids, polyphenols, and antioxidants which offer various health benefits [45]. Studies have shown that consuming different types of fruit juices is associated with improved health [[46], [47], [48], [49], [50], [51], [52]]. Additionally, the fruit juice market is a highly competitive and innovative segment within the food and beverage industry. However it holds great potential due to the increasing demand for natural and healthy products among consumers [[53], [54], [55], [56]]. Currently, consumers place significant importance on the health trend in food and beverage consumption, and this trend is expected to continue in the future [57]. As of 2023, the global fruit juice market was valued at USD 153.8 billion and is projected to reach USD 216.6 billion by 2032 [58]. Despite the industry's importance, there is limited information and data available regarding the impact of the COVID-19 pandemic on the fruit juice industry. Therefore, it is crucial to investigate and uncover the effects of the pandemic on this industry. The primary focus of this study is to investigate the effect of the COVID-19 pandemic on the fruit juice industry in Türkiye. It also seeks to identify pandemic-induced changes in companies that could be implemented in the future and discuss potential long-term consequences for the industry.

Türkiye provides an ideal case study to understand the impact of the COVID-19 pandemic on the fruit juice industry. This is because Türkiye has favorable conditions for cultivating a variety of fruits [[59], [60], [61]] and is one of the world's leading producers of fruit juice [62]. Türkiye has a high capacity for fruit production, ranking first in apricot production, second in apple production, third in sour cherry production, fourth in peach production, and sixth in grape production [63]. The Turkish fruit juice market is also dynamic and growing, with fruit juice exports reaching approximately 150 countries [64]. This study is highly valuable not only for comprehensively analyzing the impacts of the COVID-19 pandemic on the fruit juice industry in Türkiye, but also for identifying potential future developments and devising effective strategies to minimize the negative consequences of any future crises.

The findings of this study can contribute significantly to improving the efficiency and sustainability of the industry and can serve as a valuable reference for the food sector as a whole. Moreover, the study can provide insights into how other sectors can effectively respond to future disruptions and can also highlight successful practices for managing crises. In addition, the study identifies areas where the COVID-19 pandemic has led to positive transformations, which is a crucial aspect to explore since the pandemic is often associated with negative effects. Lastly, this study can also determine whether the pandemic has presented any opportunities for growth and positive changes in the fruit juice industry.

## 2 Materials and methods

The fruit juice industry in Türkiye is of considerable economic importance, serving as a vital source of income for approximately 1 million farmers and contributing to rural development. The Turkish fruit juice sector has a broad global presence, reaching over 120 countries and generating export revenues of more than USD 400 million [65]. The industry includes significant large companies.

The sample for the study consists of companies that are members of MEYED, the Turkish Fruit Juice Industry Association. MEYED is a prominent organization that represents not only fruit juice producers in the industry, but also companies that supply raw materials, packaging and equipment. The group has been a member of the European Fruit Juice Group (AIJN) and the International Federation of Fruit Juice Producers (IFU) since 2005 and 1997 respectively.

**Fig. 1:**
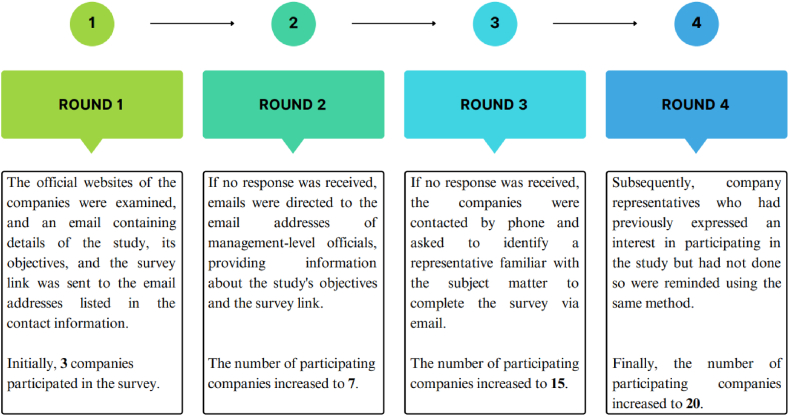
Data collection process.

The Turkish Fruit Juice Industry Association (MEYED) has a membership of 45 companies, with some specializing in fruit juice production and others providing raw materials, packaging, and equipment to the industry. From the pool of MEYED members, 26 companies produce various fruit-based beverages such as fruit juices, fruit nectars, fruit-flavored drinks, purees, and concentrates. These 26 companies are the final sample of the study. Data for the study were collected through a four-step process, as shown in Fig. 1. All companies/company representatives in the study were anonymously labeled and agreed to participate in the survey. Six companies declined to participate in the study for various reasons, leaving 20 companies that completed the survey.

The study uses a mixed-methods research design, which includes both quantitative and qualitative data. Quantitative data analysis included the use of frequency distributions, percentages and Likert scales. Furthermore, Fisher's exact test was used to investigate the statistical significance of the changes in production, sales, exports and total profits experienced by the companies in relation to company size and year of operation. Specifically, the analysis compared the pre-pandemic period (2019) with 2020, the first year of the pandemic in Türkiye, when the industry was significantly affected by the pandemic.

Qualitative data for the study was collected by asking open-ended questions of company representatives. These questions were designed to comprehensively analyze the effects of COVID-19 on companies and the fruit juice industry. The aim was to identify the positive and negative effects of the pandemic on the companies, to identify the changes brought about by the pandemic, and to determine the long-term consequences. The data was analyzed using thematic analysis. This method involves identifying and reporting on patterns (themes) in the data. Thematic analysis is a widely used method that provides a rich description of qualitative data. The six-step method of thematic analysis defined by Braun and Clarke (2006) was used in the analysis of the open-ended questions [66]. This involved becoming familiar with the data, generating initial codes, searching for themes, reviewing themes, defining and naming themes and writing the report. Word clouds were created using the NVivo program during the thematic analysis of the open-ended questions.

## 3 Results

Table 1 provides information on the companies that participated in the study, as well as the positions of the company respondents who answered the questionnaire on behalf of their respective companies.

The companies participating in the study are located in 12 different cities in Türkiye, representing a diverse range of locations within the country. The age of the companies varied considerably, with six companies operating for more than 40 years and seven companies operating for less than 20 years. Of the 20 companies, eight employ more than 250 full-time employees, indicating their status as large companies. In addition, 9 enterprises are classified as medium-sized enterprises, 2 enterprises are classified as small enterprises and one enterprise is classified as a micro-enterprise. Looking at the product lines of the participating companies, puree/concentrate was found to be the most commonly produced product, with 17 companies involved in its production. This was followed by fruit juice (12 companies), fruit nectar (10 companies), fruit drinks (10 companies), and flavored drinks (8 companies). In particular, almost all of the companies (except one brand produced by a multinational beverage company for selected countries, including Türkiye) are involved in exports, indicating the industry's overall orientation towards international markets (Table 1).

**Table 1:** Company characteristics and respondent profile.

Company	Position of Respondent	City	Age of Company	Company's Products	Company Size (Employees)	Export
C-1	Sales and Marketing Director	Konya	16	Puree/Concentrate	10–49	Yes
C-2	Quality Assurance Manager	Adana	16	Puree/Concentrate Fruit juice	50–249	Yes
C-3	Business Development Director	Antalya	29	Puree/Concentrate Fruit juice	50–249	Yes
C-4	Commercial Marketing Manager	Bursa	55	Puree/Concentrate Fruit juice Fruit nectar Fruit drink Flavored drink	250+	Yes
C-5	Country Marketing Director	İstanbul	29	Fruit juice Fruit nectar Fruit drink	250+	No
C-6	Chief Executive Officer (CEO)	Tokat	65	Puree/Concentrate Fruit juice Fruit nectar Fruit drink Flavored drink	250+	Yes
C-7	Quality Manager	Batman	9	Puree/Concentrate Fruit juice Fruit nectar Fruit drink Flavored drink	50–249	Yes
C-8	Export Specialist	Erzincan	19	Puree/Concentrate Fruit juice Fruit nectar	50–249	Yes
C-9	Quality Assurance Manager	Konya	54	Puree/Concentrate Fruit juice Fruit nectar Fruit drink Flavored drink	10–49	Yes
C-10	R&D Manager	Bursa	111	Fruit drink Flavored drink	250+	Yes
C-11	Quality Manager	Kayseri	21	Puree/Concentrate Fruit juice Fruit nectar Fruit drink	50–249	Yes
C-12	Export Manager	İstanbul	14	Fruit nectar Puree/Concentrate	250+	Yes
C-13	Export and Logistics Operations Specialist	Malatya	41	Puree/Concentrate Fruit juice	50–249	Yes
C-14	R&D Director	Adana	26	Fruit drink Flavored drink	250+	Yes
C-15	Quality Assurance Manager	Denizli	26	Puree/Concentrate	50–249	Yes
C-16	Quality Assurance Manager	Isparta	16	Puree/Concentrate	50–249	Yes
C-17	Integrated Management Systems Manager	Kayseri	30	Puree/Concentrate	50–249	Yes
C-18	Export Officer	Denizli	10	Puree/Concentrate	1–9	Yes
C-19	Export Warehouse Manager	Bursa	68	Puree/Concentrate Fruit juice Fruit nectar Fruit drink Flavored drink	250+	Yes
C-20	Export Manager	Adana	26	Puree/Concentrate Fruit juice Fruit nectar Fruit drink Flavored drink	250+	Yes

Looking at the effects of the COVID-19 pandemic on companies, it is clear that logistics activities were the most affected (with a mean score of 4.05). The supply of raw materials was also moderately affected (with a mean score of 3.25). In contrast, the impact of the pandemic on exports (with a mean score of 2.78), overall impact (with a mean score of 2.75), sales (with a mean score of 2.75), total profit (with a mean score of 2.60), production (with a mean score of 2.55), and R&D (with a mean score of 2.15) was less significant. It can therefore be said that the pandemic has had a major impact on logistics activities, a moderate impact on supply activities, and a limited impact in other areas (Table 2).

**Table 2:** Effects of the COVID-19 pandemic.

Questions	Categories	Frequency	Likert mean
How much did the COVID-19 pandemic affect your logistic activities?	Not affected at all	–	4.05
	Slightly affected	–	
	Moderately affected	5	
	Affected	9	
	Severely affected	6	
How much did the COVID-19 pandemic affect your raw material supply?	Not affected at all	3	3.25
	Slightly affected	1	
	Moderately affected	7	
	Affected	6	
	Severely affected	3	
How much did the COVID-19 pandemic affect your export activities?	Not affected at all	3	2.78
	Slightly affected	2	
	Moderately affected	11	
	Affected	2	
	Severely affected	1	
How much did the COVID-19 pandemic affect your company overall?	Not affected at all	2	2.75
	Slightly affected	3	
	Moderately affected	13	
	Affected	2	
	Severely affected	–	
How much did the COVID-19 pandemic affect your sales?	Not affected at all	2	2.75
	Slightly affected	4	
	Moderately affected	11	
	Affected	3	
	Severely affected	–	
How much did the COVID-19 pandemic affect your total profit?	Not affected at all	3	2.60
	Slightly affected	5	
	Moderately affected	9	
	Affected	3	
	Severely affected	–	
How much did the COVID-19 pandemic affect your production?	Not affected at all	5	2.55
	Slightly affected	1	
	Moderately affected	12	
	Affected	2	
	Severely affected	–	
How much did the COVID-19 pandemic affect your R&D activities?	Not affected at all	8	2.15
	Slightly affected	4	
	Moderately affected	5	
	Affected	3	
	Severely affected	–	
1 = Not affected at all, 2 = Slightly affected, 3 = Moderately affected, 4 = Affected, 5 = Severely affected			

The COVID-19 pandemic has posed significant challenges for companies, resulting in several negative effects. Logistics activities have been most affected, with 17 out of 20 companies experiencing various logistical problems as a result of the pandemic. In addition to this, companies have also faced cost increases in various categories of expenditure, as well as challenges with raw material supply. Eight companies experienced financial difficulties and another eight had to postpone investments due to pandemic. Four companies temporarily stopped production, and four others experienced a decrease in demand for their products. One company reported an increase in debt as a result of the pandemic. It can therefore be concluded that the main challenges faced by companies with COVID-19 are related to logistics, cost increases and raw material issues (Table 3).

**Table 3:** Negative effects of COVID-19.

Negative Effects	Categories	Frequency	Percent
We have experienced various problems related to logistics.	Yes	17	85.0 %
	No	3	15.0 %
We had significant cost increases in various expense categories.	Yes	15	75.0 %
	No	5	25.0 %
We have experienced difficulties in the supply of raw materials	Yes	14	70.0 %
	No	6	30.0 %
We have experienced various financial problems.	Yes	8	40.0 %
	No	12	60.0 %
We had to postpone various investments.	Yes	8	40.0 %
	No	12	60.0 %
We temporarily stopped our production.	Yes	4	20.0 %
	No	16	80.0 %
Demand for our products declined.	Yes	4	20.0 %
	No	16	80.0 %
We experienced an increase in debt.	Yes	1	5.0 %
	No	19	95.0 %

The effects of the COVID-19 pandemic on companies in Türkiye were analyzed by comparing their production, sales, exports, and total profits in the pre-pandemic year of 2019 with those in 2020, the first year in which the pandemic was strongly felt in the country.

According to the data, in 2020, production decreased for eight companies, sales decreased for seven companies, exports decreased for six companies and total profits decreased for eight companies. In contrast, the analysis showed that in 2020, the year of the pandemic, production, sales, and total profits increased for nine companies, while exports increased for seven companies. Although some companies were adversely affected by COVID-19, a significant number of companies experienced growth in their production, sales, exports, and total profits as a result of the pandemic. Notably, no significant relationship (p > 0.05) was found between the changes in production, sales, exports, total profit, and the size of the company using Fisher's exact test (Table 4).

**Table 4:** Comparison of company performance between pre-pandemic period (2019) and first year of COVID-19 pandemic (2020) by Size.

Variables	Categories	Company Size			p-value
		Small^a^ (n = 3)	Medium (n = 9)	Large (n = 8)	
Production	Decreased	1	3	4	0.734
	Unchanged	1	1	2	
	Increased	1	5	2	
Sale	Decreased	1	3	3	0.924
	Unchanged	1	1	2	
	Increased	1	5	3	
Export^b^	Decreased	1	3	2	0.316
	Unchanged	1	1	4	
	Increased	1	5	1	
Total Profit	Decreased	1	3	4	0.337
	Unchanged	1	0	2	
	Increased	1	6	2	

a One micro-enterprise is also included in the small category.

b The total number of enterprises in this section is 1 less than the others because 1 enterprise does not export.

The analysis showed that there was no significant relationship (p > 0.05) between the changes in production, sales, exports, total profit, and the number of years the companies had been in operation, according to Fisher's exact test (Table 5).

**Table 5:** Comparison of company performance between the pre-pandemic Period (2019) and the first year of the COVID-19 pandemic (2020) by year of operation.

Variables	Categories	Year of operation		p-value
		1–20 years (n = 7)	20+ years (n = 13)	
Production	Decreased	2	6	0.205
	Unchanged	3	1	
	Increased	2	6	
Sale	Decreased	2	5	0.848
	Unchanged	2	2	
	Increased	3	6	
Export^a^	Decreased	2	4	1.000
	Unchanged	2	4	
	Increased	3	4	
Total Profit	Decreased	2	6	0.818
	Unchanged	1	2	
	Increased	4	5	

a The total number of enterprises in this section is 1 less than the others because 1 enterprise does not export.

Companies have taken several steps to mitigate the negative effects of the COVID-19 pandemic. The vast majority of companies (90.0 %) have taken steps to ensure employee health and food safety by introducing new health and safety protocols in response to the pandemic. In addition, 80.0 % of companies introduced remote working at some point during the pandemic, and 60.0 % have established new supplier relationships. While a smaller number of companies have launched new products (20.0 %) or reduced prices (10.0 %), a significant number (30.0 %) have increased their online sales channels. No companies declared bankruptcy, and one company restructured its bank loans. Overall, the data show that companies are taking active measures to adapt to the challenges posed by COVID-19, including prioritizing health and safety, exploring new supplier relationships, and adopting online sales channels. These findings suggest that companies are taking proactive steps to adapt and ensure long-term survival in the face of the ongoing pandemic (Table 6).

**Table 6:** Companies' strategies to mitigate the negative effects of COVID-19.

Strategies	Categories	Frequency	Percent
We introduced new health and safety protocols.	Yes	18	90.0 %
	No	2	10.0 %
We introduced remote working.	Yes	16	80.0 %
	No	4	20.0 %
We started working with new suppliers.	Yes	12	60.0 %
	No	8	40.0 %
We increased our online sales channels.	Yes	6	30.0 %
	No	14	70.0 %
We introduced new products to the market.	Yes	4	20.0 %
	No	16	80.0 %
We reduced our prices.	Yes	2	10.0 %
	No	18	90.0 %
We restructured our bank loans.	Yes	1	5.0 %
	No	19	95.0 %
We filed for bankruptcy.	Yes	0	0.0 %
	No	20	100.0 %

The results show that the vast majority of companies (85.0 %) reported a positive situation compared to the period of intense impact of the pandemic, with no companies reporting that their situation had worsened. These results suggest that the fruit juice industry has been able to successfully overcome the challenges posed by the pandemic. In addition, the majority of companies (80.0 %) expressed confidence in their future performance, indicating a positive outlook for the industry in the upcoming period. This optimistic perspective highlights the resilience of the industry highlights the industry's resilience and its ability to adapt to new challenges (Table 7).

**Table 7:** Current situation and future of the sector after COVID-19.

Questions	Categories	Frequency	Percent
How would you evaluate the current overall situation/performance of your company, compared to the period when the pandemic had intense effects?	Very bad	–	0.0 %
	Bad	–	0.0 %
	Same	3	15.0 %
	Good	14	70.0 %
	Very good	3	15.0 %
How do you anticipate your company's performance and overall situation will compare to the present in the upcoming period?	Very bad	–	0.0 %
	Bad	1	5.0 %
	Same	3	15.0 %
	Good	10	50.0 %
	Very good	6	30.0 %

To obtain more comprehensive and detailed results on the impact of the pandemic on the industry, open-ended questions were asked of the participating companies. One such question was, “Has the COVID-19 pandemic resulted in any significant changes in your company that are being implemented for the future? If so, what are these changes?” Of the total number of companies surveyed, 6 responded in the affirmative, while 14 reported no significant changes (Table 8).

**Table 8:** Significant changes for future implementation.

Question	Categories	Frequency	Percent
Has the COVID-19 pandemic resulted in any significant changes in your company that are being implemented for the future?	Yes	6	30.0 %
	No	14	70.0 %

**Fig. 2:**
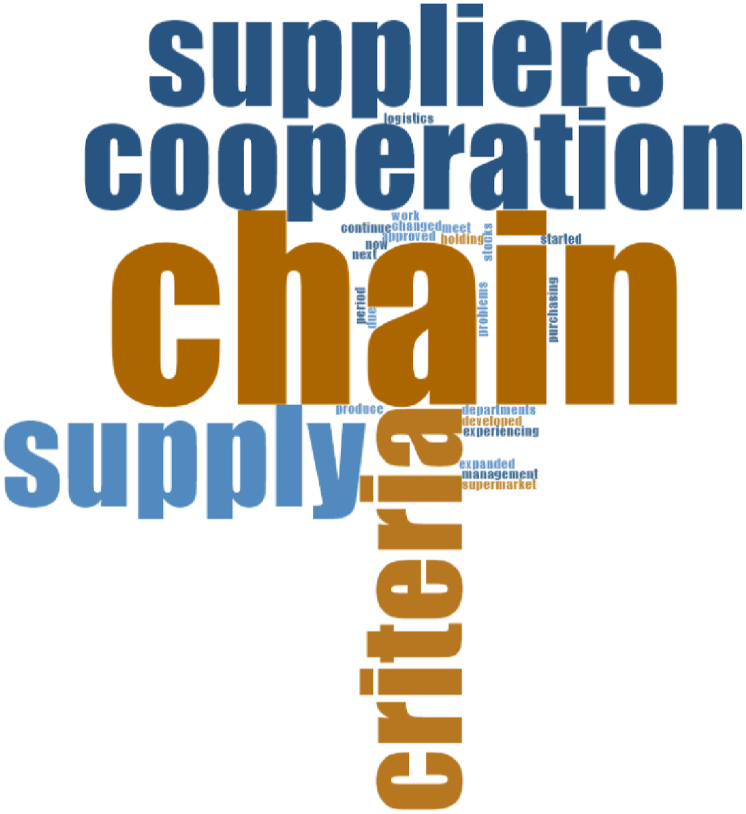
Word cloud describing the changes in the industry caused by the COVID-19 pandemic.

After analyzing the responses of the companies that reported significant changes due to the COVID-19 pandemic, a word cloud was created to illustrate the most commonly cited changes (Fig. 2).

The responses received from companies were subjected to thematic analysis, combining similar responses to identify key themes. As a result, the themes of cooperation and supply chain management emerged (Table 9).

**Table 9:** Themes identified on the changes caused by the COVID-19 pandemic in the industry.

Themes	Answers
Cooperation	- We have developed our cooperation with supermarket chains to continue in the next period.
Supply Chain Management	- We have changed our criteria for approved suppliers and are now working with suppliers who meet these criteria. - We have expanded our logistics and purchasing departments due to the problems we have experienced in the supply chain. - We have started to produce by holding more stock.

Following a thematic analysis of the responses received from companies, supply chain management emerged as the most prominent theme. Significant changes were observed in this area, such as companies changing their supplier selection criteria, expanding their logistics and purchasing departments, and increasing their stock levels. Another theme that emerged was cooperation, as some companies had established partnerships with supermarket chains, a change that is likely to continue in the future.

When asked whether the COVID-19 pandemic had a positive impact on their company, 16 out of 20 companies responded positively, with the remaining 4 companies reporting no significant impact. It is noteworthy that 80 % of companies reported a positive impact, suggesting that some companies may have found opportunities to adapt and thrive amidst the challenges of the pandemic (Table 10).

**Table 10:** Positive impacts of the COVID-19 pandemic for companies.

Question	Categories	Frequency	Percent
Has the COVID-19 pandemic had a positive impact on your company?	Yes	16	80.0 %
	No	4	20.0 %

**Fig. 3:**
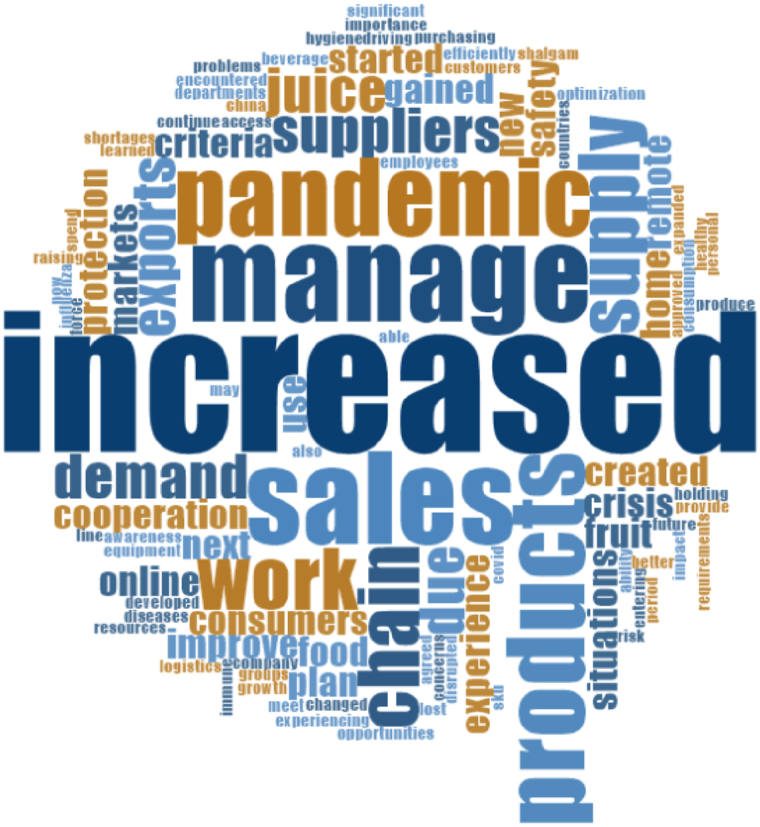
Word cloud describing the positive effects of the COVID-19 pandemic for companies.

After analyzing the responses of the companies that reported positive effects due to the COVID-19 pandemic, a word cloud was created to illustrate the most commonly cited positive effects (Fig. 3).

The responses received from companies were subjected to thematic analysis, combining similar responses to identify key themes. As a result, the themes of demand/sale, export, food safety and management emerged (Table 11).

**Table 11:** Themes identified on the positive impacts of the COVID-19 pandemic for companies.

Themes	Answers
Demand/Sale	- As beverage consumption at home increased during the pandemic, our fruit juice sales also increased. - As consumers spent more time at home, sales of some our product groups increased. - Our customers demanded more due to the risk of supply shortages and concerns that they would not be able to access products. - Our online sales increased in line with the growth of online commerce during the pandemic. - Our sales increased as demand for healthy products increased. - Our sales have increased as consumers believe that fruit juices support the immune system and that products such as shalgam juice provide protection against diseases such as influenza.
Export	-Unexpected production schedules and a disrupted supply chain created opportunities for us and our exports to the US increased. -We have increased our exports by entering new markets (countries) where China lost markets due to the pandemic.
Food Safety	- It has had a significant impact on raising food safety awareness in our company. - Our employees have a better understanding of hygiene requirements and the importance of using personal protective equipment.
Management	- The COVID-19 pandemic created a driving force to improve our SKU optimization - We agreed and started working with new suppliers. -We further improved our ability to use and manage resources more efficiently. - We gained experience of how to manage and plan for crisis situations. - We gained experience of how to manage the next crisis or pandemic situation that may arise in the future. - We have learned how to work remotely and how to manage remote working well.

Analysis of the Demand/Sale theme shows that the COVID-19 pandemic has had a significant impact on companies, resulting in positive effects on demand and sales. In particular, many companies have reported an increase in at-home beverage consumption at home due to increased time spent indoors during the pandemic. This trend has had a positive impact on fruit juice sales, as consumers perceive these beverages as healthy and immune-boosting. Consumers also stockpiled more food at the onset of the pandemic out of concern for possible shortages, which also benefited fruit juice companies. In addition, the growth of online retailing during the pandemic led to an increase in online sales for many companies.

The COVID-19 pandemic has had a positive effect on the export activities of some companies. Despite production and supply chain disruptions around the world, some companies have seized new opportunities and expanded their export markets. In particular, the pandemic has caused China's exports to shrink, creating space for some companies to enter and thrive in new markets.

In addition, the pandemic has highlighted the importance of food safety, prompting many companies to invest in employee training and education. As a result, employees are more aware of hygiene practices, better equipped to use protective equipment and more knowledgeable about food safety issues.

Regarding management, the pandemic has also brought about positive changes. Companies have optimized their stock keeping units (SKUs) and expanded their supplier networks by partnering with new suppliers. The pandemic has also enabled companies to become more efficient in resource management, adapt to remote working, and develop crisis management skills. Consequently, many companies have increased their flexibility and learned how to better manage potential bottlenecks and crises.

Although the COVID-19 pandemic has had negative effects, it is worth noting that fruit juice companies have experienced some positive impacts.

The impact of the COVID-19 pandemic on companies' goals and potential was assessed by asking whether the pandemic had prevented them from achieving any of their objectives. Ten out of the twenty companies surveyed responded affirmatively, while the remaining ten responded negatively (Table 12).

**Table 12:** The goals and potentials that companies were unable to achieve due to the COVID-19 pandemic.

Question	Categories	Frequency	Percent
Has your company been unable to achieve any of its goals or potentials due to the COVID-19 pandemic?	Yes No	10 10	50.0 % 50.0 %

**Fig. 4:**
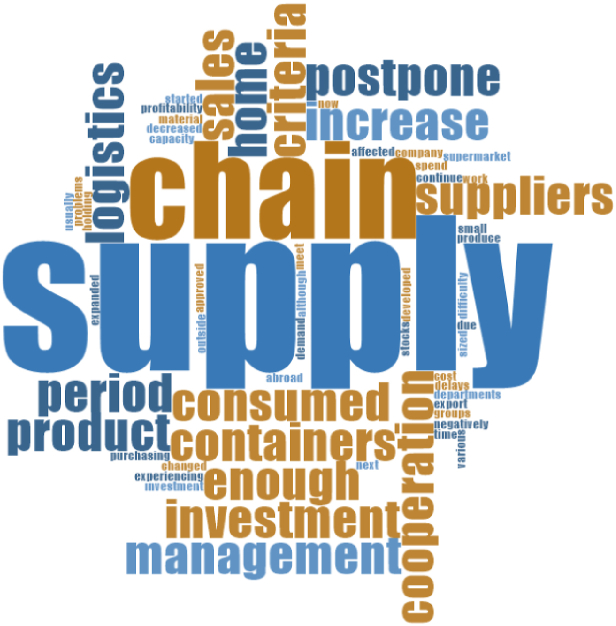
Word cloud describing the goals/potentials hindered by the COVID-19 pandemic in companies.

After analyzing the responses of the companies that reported goals and potentials that were hindered by the COVID-19 pandemic, a word cloud was created to illustrate the most commonly cited goals and potentials (Fig. 4).

The responses received from companies were subjected to thematic analysis, combining similar responses to identify key themes. As a result, the themes of investment, sales and supply chain management emerged (Table 13).

**Table 13:** Themes identified on the targets/potentials prevented by the COVID-19 pandemic in companies.

Themes	Answers
Investment	- Our investment in capacity increase has been postponed. - Our various investments have been postponed.
Sales	- Sales of our small-sized product groups, which are typically consumed outside the home, have decreased as consumers spend more time at home.
Supply Chain Management	- Although there was demand for our products from abroad, we were unable to export for a period due to insufficient container supply. - Our company's profitability was negatively affected by delays in material supply. - We experienced an increase in logistics costs due to difficulties in supplying enough containers.

The COVID-19 pandemic has presented a range of challenges for companies in the field of investment. Some have had to postpone investment plans, such as increasing capacity, which has hindered their ability to reach certain targets.

Although some companies have seen a rise in sales during due to the pandemic, smaller product groups that are usually consumed outside of the home have experienced a decrease in sales, as more consumers spend time at home.

Supply chain management has also faced significant challenges during the pandemic. Companies have faced challenges in sourcing containers, resulting in disruptions to planned exports. In addition, logistics costs have increased due to the limited availability of containers. Furthermore, delays in material supply have had a negative impact on company profitability.

Companies were asked whether they thought the COVID-19 pandemic would have a long-term impact on the fruit juice industry. Of the twenty companies surveyed, seven responded positively and the remaining thirteen responded negatively (Table 14).

**Table 14:** Long-term impact of the COVID-19 pandemic on industry.

Question	Categories	Frequency	Percent
Do you think that the COVID-19 pandemic will have a long-term impact on the fruit juice industry?	Yes No	7 13	35.0 % 65.0 %

**Fig. 5:**
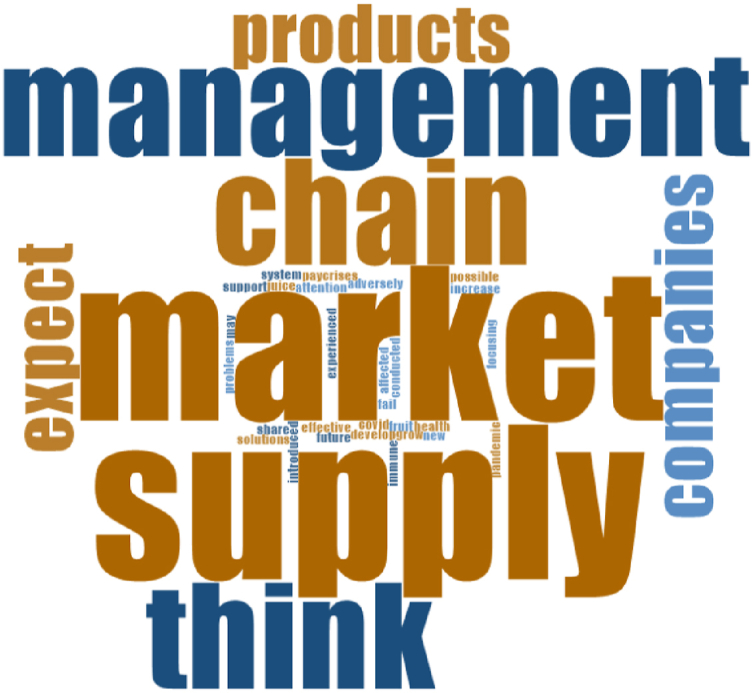
Word cloud describing the long-term impact of the COVID-19 pandemic on industry.

Companies that responded in the affirmative regarding the long-term impact of the COVID-19 pandemic for the fruit juice industry were asked to elaborate on their responses. A word cloud was created to illustrate the most commonly cited impacts (Fig. 5).

The responses received from companies were subjected to thematic analysis, combining similar responses to identify key themes. As a result, the themes of market and supply chain management emerged (Table 15).

**Table 15:** Themes identified on the long-term impact of the COVID-19 pandemic.

Themes	Answers
Market	- We anticipate that the R&D conducted during the COVID-19 pandemic will lead to the launch of new products with a stronger health focus. - We expect the market share of products that support the immune system to increase. - We expect the fruit juice market to continue to grow.
Supply Chain Management	- We expect that companies that fail to develop effective supply chain management solutions will be adversely affected by supply problems that may recur in possible future crises. - We expect companies to pay more attention to supply chain management.

Long-term changes in the fruit juice market are expected by some companies as a result of the COVID-19 pandemic. These changes include further growth in the juice market, the introduction of new health-oriented products, and an increased emphasis on health trends in the market.

The pandemic is also expected to have a long-term impact on supply chain management. In particular, it is expected that companies will place greater emphasis on effective supply chain management, and those that fail to do so and cannot develop effective solutions to supply chain problems will be negatively affected by future crises.

## 4 Discussion and conclusion

The dominant structure of the industry consists of medium and large enterprises engaged in export activities. The COVID-19 pandemic had a significant impact on companies' logistics activities, but only a moderate impact on supply activities. The pandemic had a more limited impact on exports, sales, production, total profit, and R&D activities.

Comparing 2020 with 2019, some companies in the industry experienced decreases in production, sales, exports, and total profit, while others experienced increases in these variables. The changes were independent of company size and year of operation, suggesting that the impact of the pandemic on each company was unique.

The COVID-19 pandemic had a number of negative impacts on companies, with logistics being the most affected area. Companies faced increased costs and supply problems due to shortages of raw materials. Half of the companies surveyed were unable to meet their objectives due to the pandemic, particularly in terms of investment and supply chain management. Companies faced difficulties in obtaining containers, leading to increased logistics costs and a reduction in targeted and planned exports.

In response to the COVID-19 pandemic, companies in the Turkish fruit juice industry have taken various measures to mitigate its effects and minimize the damage they have suffered. One of the most important measures taken by the companies was the establishment and implementation of new health and safety protocols aimed at protecting the health of employees and ensuring food safety. Many companies have also moved to remote working and started working with new suppliers to reduce and prevent supply problems.

The majority of companies in the industry consider their situation to be better then it was when the impact of the pandemic was more severe. Despite the challenges faced, companies remain generally optimistic about the future, both at sector and company level.

For some companies, the pandemic has led to significant changes that will be implemented from now on. These include developing cooperation with supermarkets, changing the criteria for approved suppliers, working with new suppliers, expanding logistics and purchasing departments, and increasing stock levels. These changes reflect the companies’ adaptation to the new normal and their efforts to mitigate the impact of potential future crises.

One remarkable finding is that 80.0 % of the surveyed companies reported positive effects from the COVID-19 pandemic. This is noteworthy because it shows that, despite the pandemic's predominantly negative effects, it can have positive impacts on some industries. Companies have experienced increased demand and sales for certain products, and the global export and supply chain disruptions have created opportunities for some companies, resulting in positive impacts on their exports. The pandemic has had a significant impact on food safety practices, raising awareness and improving them in many companies. Some companies have also turned the crisis into an opportunity by expanding their supplier networks and improving their crisis management skills.

Some companies are also anticipating the long-term impact of the COVID-19 pandemic. They expect that the juice market will continue to grow after the pandemic and that new health-focused products will be introduced, with a greater emphasis on health trends in the juice market. In addition, supply chain management is expected to become an area of greater focus for companies in the post-pandemic period.

In brief, the COVID-19 pandemic has had both negative and positive effects on the Turkish fruit juice industry. While certain areas, such as logistics and supply, have been significantly affected, the industry as a whole has not been adversely affected to a great extent. However, it has highlighted vulnerabilities in certain areas. To be more resilient to potential crises, industry players can expand their supply channels, optimize stock management, and increase flexibility in logistics processes. Additionally, digital marketing, online sales, and distribution can help companies maintain their business operations in times of crisis. Overall, the pandemic has created opportunities for the industry to adapt and grow in the long term.

Given the challenges faced by the industry in logistics and supply chain management during the COVID-19 pandemic, future research should focus on these areas in greater detail. Studies that focus on successful supply chain management during crises, such as COVID-19, and minimizing disruptions can make significant contributions to the literature.

Considering that the COVID-19 pandemic, often associated with negative effects, has unexpectedly yielded several positive outcomes for the fruit juice industry, it is also beneficial for future studies to delve into these positive findings. Moreover, future research should explore avenues through which crisis periods like COVID-19 can be leveraged as opportunities to foster positive impacts. By shifting focus towards these aspects, researchers can provide valuable insights into harnessing adversity for growth and innovation within the industry.

## Funding statement

This research received no funding.

## Ethics statement

All companies/company representatives in the study were anonymously labeled and agreed to participate in the survey. All participants provided informed consent to participate in the study.

## Data availability statement

The data that support the findings of this study are available on request from the corresponding author. The data are not publicly available due to privacy or ethical restrictions.

## CRediT authorship contribution statement

**Berkay Keskin:** Writing – review & editing, Writing – original draft, Visualization, Validation, Supervision, Software, Resources, Methodology, Investigation, Formal analysis, Data curation, Conceptualization.

## Declaration of competing interest

The authors declare that they have no known competing financial interests or personal relationships that could have appeared to influence the work reported in this paper.

## Appendix A Supplementary data

Multimedia component 1 Questionnaire Form
