# Supplementary material for: The effect of COVID-19 pandemic on the fruit juice industry: Insights from Türkiye
Source: Heliyon. 2024 Apr 9;10(9):e29406. doi: 10.1016/j.heliyon.2024.e29406 (PMC11066138; doi:10.1016/j.heliyon.2024.e29406)
Supplement: Multimedia component 1 [file mmc1.pdf]

Firma Anket Formu

Sayın Katılımcı, bu anket formu Covid-19 pandemisinin Türkiye meyve suyu endüstrisine etkilerini ortaya koymayı amaçlamaktadır. Anket formu meyve suyu/püre/konsantre üreten firmalara uygulanmaktadır. Covid-19 pandemisinin etkileri ile ilgili soruları değerlendirirken lütfen pandeminin en yoğun etkilerinin görüldüğü 2020 yılını dikkate alarak soruları cevaplandırınız. Sorulara vereceğiniz cevaplar gizli tutulacak ve tüm katılımcılardan elde edilen veriler toplu olarak değerlendirilecektir.

Anket formunda veri eksiğinin olabileceği alanlar düşünülerek bazı soruların cevaplanmaması halinde de anketin bitirilebilmesi sağlanmıştır. Ancak tüm soruları cevaplamanız bu araştırmanın istenilen hedeflerle tamamlanması için büyük katkıda bulunacaktır. Anket formunu bitirdikten sonra en altta bulunan "Gönder" düğmesini tıklayarak anketi tamamlayabilirsiniz.

Bu araştırmaya katılmayı kabul etmemeniz ya da araştırmadan ayrılmanız durumunda karşı karşıya kalacağınız herhangi bir olumsuz sonuç bulunmamaktadır. Çalışma için size ödeme yapılmayacak, sizden de ücret talep edilmeyecektir.
Bu araştırmada yer almak tümüyle sizin isteğinize bağlıdır. Araştırmada yer almayı reddedebilirsiniz ya da başladıktan sonra yarıda bırakabilirsiniz. Bu araştırmanın sonuçları bilimsel amaçlarla kullanılacaktır. Araştırmadan çekilmeniz halinde, sizle ilgili veriler kullanılmayacaktır. Ancak veriler bir kez anonimleştikten sonra araştırmadan çekilmeniz mümkün olmayacaktır. Sizden elde edilen tüm bilgiler gizli tutulacak, araştırma yayınlandığında da varsa kimlik bilgilerinizin gizliliği korunacaktır.

Yukarıda yer alan ve araştırmaya başlanmadan önce gönüllülere verilmesi gereken bilgileri içeren metni okudum (ya da sözlü olarak dinledim). Eksik kaldığını düşündüğüm konularda sorularımı araştırmacılara sordum ve doyurucu yanıtlar aldım. Yazılı ve sözlü olarak tarafıma sunulan tüm açıklamaları ayrıntılarıyla anladığım kanısındayım. Çalışmaya katılmayı isteyip istemediğim konusunda karar vermem için yeterince zaman tanındı.

Bu koşullar altında, araştırma kapsamında elde edilen şahsıma ait bilgilerin bilimsel amaçlarla kullanılmasını, gizlilik kurallarına uyulmak kaydıyla sunulmasını ve yayınlanmasını, hiçbir baskı ve zorlama altında kalmaksızın, kendi özgür irademle kabul ettiğimi beyan ederim.

Araştırmaya olan katkınız için teşekkür ederim.

Berkay KESKİN (bkeskin@ankara.edu.tr)
Ankara Üniversitesi Ziraat Fakültesi
Tarım Ekonomisi Bölümü

* Zorunlu soruyu belirtir

1. 1- Firma ismi *

____

2. 2- Anketi dolduran kişinin şirketteki görevi

3. 3-Firmada Tam Zamanlı Olarak Çalışan Toplam Personel Sayısı

4. 4- Firma kuruluş yeri

5. 5- Firma kuruluş yılı

6. 6- Ürettiğiniz ürün tipleri nelerdir? (Birden fazla işaretleyebilrsiniz.)

   Uygun olanların tümünü işaretleyin.

   - [ ] Meyve suyu (%100)
   - [ ] Meyve nektarı (%25-99)
   - [ ] Meyveli içecek (%10-24)
   - [ ] Aromalı içecek (%0-9)
   - [ ] Püre/Konsantre/Püre-Konsantre

7. 7- Yukarıdaki soruda belirtmiş olduğunuz ürün tipleri için ürettiğiniz toplam ürün ne kadardır (Ton cinsinden belirtiniz.)

8. 8- Firma olarak 2019 yılı karınız toplam ne kadar? ($, € ya da TL cinsinden yazabilirsiniz. Lütfen birimi ekleyiniz.)

9. 9- İhracat yapıyor musunuz?

Yalnızca bir şıkkı işaretleyin.

- Evet
- Hayır

10. 10- İhracat yapıyorsanız kaç ülkeye yapıyorsunuz? (Yapmıyorsanız soruyu boş bırakınız.)

____

11. 11- Covid-19 pandemisi firmanızı ne ölçüde etkiledi?

Yalnızca bir şıkkı işaretleyin.

- Hiç etkilemedi
- Az etkiledi
- Orta derecede etkiledi
- Epey etkiledi
- Çok fazla etkiledi

12. 12- Covid-19 pandemisi üretiminizi ne ölçüde etkiledi?

Yalnızca bir şıkkı işaretleyin.

- Hiç etkilemedi
- Az etkiledi
- Orta derecede etkiledi
- Epey etkiledi
- Çok fazla etkiledi

13. 13- Covid-19 pandemisi satışlarınızı ne ölçüde etkiledi?

Yalnızca bir şıkkı işaretleyin.

- Hiç etkilemedi
- Az etkiledi
- Orta derecede etkiledi
- Epey etkiledi
- Çok fazla etkiledi

14. 14-Covid 19 pandemisi ihracatınızı ne ölçüde etkiledi? (İhracat yapmıyorsanız boş bırakınız.)

Yalnızca bir şıkkı işaretleyin.

- Hiç etkilemedi
- Az etkiledi
- Orta derecede etkiledi
- Epey etkiledi
- Çok fazla etkiledi

15. 15- Covid-19 pandemisi toplam kârınızı ne ölçüde etkiledi?

Yalnızca bir şıkkı işaretleyin.

- Hiç etkilemedi
- Az etkiledi
- Orta derecede etkiledi
- Epey etkiledi
- Çok fazla etkiledi

16. 16- Covid 19 pandemisi hammadde tedariğinizi ne ölçüde etkiledi?

Yalnızca bir şıkkı işaretleyin.

- Hiç etkilemedi
- Az etkiledi
- Orta derecede etkiledi
- Epey etkiledi
- Çok fazla etkiledi

17. 17- Covid 19 pandemisi lojistik faaliyetlerinizi ne ölçüde etkiledi?

Yalnızca bir şıkkı işaretleyin.

- Hiç etkilemedi
- Az etkiledi
- Orta derecede etkiledi
- Epey etkiledi
- Çok fazla etkiledi

18. 18- Covid 19 pandemisi AR-GE çalışmalarınızı ne ölçüde etkiledi?

Yalnızca bir şıkkı işaretleyin.

- Hiç etkilemedi
- Az etkiledi
- Orta derecede etkiledi
- Epey etkiledi
- Çok fazla etkiledi

19. 19- Covid-19 pandemisine bağlı olarak personel sayınızda nasıl bir değişim oldu?

Yalnızca bir şıkkı işaretleyin.

- Önemli ölçüde azaldı (%30'dan fazla)
- Bir ölçüde azaldı (%1-30 arası)
- Değişmedi
- Bir ölçüde arttı (%1-30 arası)
- Önemli ölçüde arttı (%30'dan fazla)

20. 20- Covid-19 pandemisine bağlı olarak aşağıdaki olumsuzluklardan hangisi/hangileriyle karşılaştınız? (Birden fazla işaretleyebilirsiniz.)

Uygun olanların tümünü işaretleyin.

- [ ] Geçici olarak üretimi durdurma
- [ ] Hammadde temininde güçlükler
- [ ] Çeşitli maliyetlerde belirgin artış
- [ ] Çeşitli finansal problemler
- [ ] Çeşitli lojistik problemleri
- [ ] Borçlarda artış
- [ ] Yatırımların ertelenmesi
- [ ] Ürünlerimize olan talepte azalma
- [ ] Hiçbiri
- [ ] Diğer: ______________________

Aşağıdaki sorularda pandemi öncesi dönemle pandeminin ilk yılı olan 2020 yılı arasında karşılaştırma yapmak hedeflenmektedir. Bu nedenle aşağıdaki soruları pandeminin ilk yılı olan 2020 yılını temel alarak, 2019 yılıyla karşılaştırma yaparak cevaplayınız.

21. 21- 2020 yılında üretiminizde nasıl bir değişiklik oldu?

Yalnızca bir şıkkı işaretleyin.

- Önemli ölçüde azaldı (%30’dan fazla)
- Bir ölçüde azaldı (%1-30 arası)
- Değişmedi
- Bir ölçüde arttı (%1-30 arası)
- Önemli ölçüde arttı (%30’dan fazla)

22. 22- 2020 yılında satışlarınızda nasıl bir değişiklik oldu?

Yalnızca bir şıkkı işaretleyin.

- Önemli ölçüde azaldı (%30’dan fazla)
- Bir ölçüde azaldı (%1-30 arası)
- Değişmedi
- Bir ölçüde arttı (%1-30 arası)
- Önemli ölçüde arttı (%30’dan fazla)

23. 23- 2020 yılında ihracatınızda nasıl bir değişiklik oldu?

Yalnızca bir şıkkı işaretleyin.

- Önemli ölçüde azaldı (%30’dan fazla)
- Bir ölçüde azaldı (%1-30 arası)
- Değişmedi
- Bir ölçüde arttı (%1-30 arası)
- Önemli ölçüde arttı (%30’dan fazla)

24. 24- 2020 yılında toplam kârınızda nasıl bir değişiklik oldu?

Yalnızca bir şıkkı işaretleyin.

- Önemli ölçüde azaldı (%30'dan fazla)
- Bir ölçüde azaldı (%1-30 arası)
- Değişmedi
- Bir ölçüde arttı (%1-30 arası)
- Önemli ölçüde arttı (%30'dan fazla)

25. 25- Covid-19 pandemisi ile mücadele ederken herhangi bir dönemde aşağıdakilerden hangisini/ hangilerini uyguladınız? (Birden fazla işaretleyebilirsiniz.)

Uygun olanların tümünü işaretleyin.

- [ ] Personel sayımızı azalttık
- [ ] Uzaktan çalışma uyguladık
- [ ] Banka kredilerimizi yeniden yapılandırdık
- [ ] Yeni tedarikçilerle çalışmaya başladık
- [ ] Piyasaya yeni ürünler sunduk
- [ ] Online satış imkanımızı arttırdık
- [ ] Ürün fiyatlarında indirim yaptık
- [ ] Yeni sağlık ve güvenlik protokolleri uygulamaya koyduk.
- [ ] Konkordato ilan ettik
- [ ] Hiçbiri
- [ ] Diğer: ______________________________

26. 26- Pandeminin yoğun etkilerinin olduğu dönemle kıyasladığınızda şu anda genel anlamda firmanızın durumunu/performansını nasıl değerlendirirsiniz?

Yalnızca bir şıkkı işaretleyin.

- Çok kötü
- Kötü
- Aynı
- İyi
- Çok iyi

27. 27- Bundan sonraki 1 yıl içerisinde firmanızın genel durumunun/performansının şimdiye kıyasla nasıl olacağını öngörmektesiniz?

Yalnızca bir şıkkı işaretleyin.

- Çok kötü
- Kötü
- Aynı
- İyi
- Çok iyi

28. 28- Pandeminin ardından firmanızda herhangi bir alanda (Üretim- Operasyonel-Ar-ge v.b.) bundan sonrası için uygulanacak önemli bir değişiklik oldu mu? Olduysa lütfen belirtiniz.

29. 29- Sizce pandemi sürecinin firmanız için herhangi bir OLUMLU etkisi oldu mu? Olduysa lütfen belirtiniz.

30. 30- Sizce meyve suyu endüstrisi için Covid-19 pandemisinin uzun dönemli sonuçları neler olacak?

31. 31- Covid-19 pandemisinin yaşanması firma olarak hangi hedeflerinizin/potansiyellerinizin gerçekleşmesini engelledi? Varsa lütfen belirtiniz.

32. 32- Konuya ya da ankete ilişkin herhangi bir eklemek istediğiniz varsa lütfen belirtiniz.

33. Gerektiğinde anketle ilgili size ulaşabilmem için lütfen mail adresinizi belirtiniz.

Çalışmaya katkınız için içtenlikle teşekkür eder, iyi çalışmalar dilerim.

Bu içerik Google tarafından oluşturulmamış veya onaylanmamıştır.

Google Formlar
